# Effects of Visual Display Unit on Ocular Accommodation in Young Adults

**DOI:** 10.22599/bioj.396

**Published:** 2025-04-29

**Authors:** Vishal Biswas, Roshni Majumder

**Affiliations:** 1 Noida International University, IN; 2 Department of Optometry, School of Allied Health Sciences, Noida International University, IN

**Keywords:** accommodation, gaming, Near point of accommodation, iPad, digital device

## Abstract

**Purpose:**

To evaluate the impact of gaming on ocular accommodative parameters.

**Methods:**

A comparative and experimental study was conducted among non-orthoptic university students for a duration of six months from September 2023 to March 2024. After a half-hour of gaming, the subjects’ accommodative parameters were examined and compared. The accommodative parameters before and after iPad gaming were compared.

**Results:**

The study included 80 participants (mean age 22.96 ± 2.23 years; 50 males, 30 females). Post gaming near-point of accommodation decreased (right eye: 10.75D to 7.15D, p < 0.001), Negative Relative Accommodation (NRA) increased from 2.64 ± 0.23 DS to 2.92 ± 0.37 DS, whereas Positive Relative Accommodation (PRA) decreased from –2.46 ± 0.41 DS to –1.78 ± 0.31 DS. The Monocular Estimation Method (MEM) values showed accommodative lag (right eye: +0.46 ± 0.15 DS to +1.24 ± 0.26 DS, p < 0.001). Monocular Accommodative Facility (MAF) and Binocular Accommodative Facility (BAF) exhibited decrease in facility (MAF; right eye: 10.34 to 4.54 cpm, BAF: 10.65 to 4.90 cpm, p < 0.001).

**Conclusion:**

This study shows 30 minutes of gaming using digital devices leads to decrease in accommodative parameters, potentially causing ocular fatigue and binocular vision anomalies in young individuals.

## Introduction

All age groups have seen significant increases in the use of digital devices in recent years, to the point that daily heavy use for social and professional purposes is now considered normal. A variety of ocular and visual symptoms have been referred to as two basic groups, dry eye syndrome and binocular vision anomalies (Sheppard and Wolffsohn, 2018). Small electronic devices such as tablets, iPads, and smartphones have become more popular recently because they make it easier and faster to complete a variety of tasks like information retrieval via the internet and schedule management without being constrained by time or location (van Kraalingen, 2023; Yalçın *et al*., 2022). As a result, close work with smartphone use, which requires more adaptation than other forms of near work, such as reading books and seeing computer monitors, may cause alterations in visual function. The increasing number of patients who are seeking therapeutic assistance for ocular disorders are related with increased usage of smart gadgets (Kang *et al*., 2021). The handheld smart devices differ from desktop to laptop in numerous ways, including viewing position and distance, brightness, screen size, and usage habits. In particular, the viewing distance for smart gadgets is quite near to that of computers, which might cause eye strain owing to accommodation and convergence (Tosha *et al*., 2009). Near-field work may induce iris constriction and accommodative spasms, which can lead to impaired accommodative functioning. This deterioration of accommodative abilities may have a negative impact on ocular tiredness (Siderov *et al*., 2001).

Previous studies have shown changes to accommodation (lower amplitude and increased latency), blink rate, tear function and dry eye symptoms when assessed before and after smartphone or tablet use (Golebiowski *et al*., 2020; Nayak *et al*., 2020; Padavettan *et al*., 2021). However, smart device activities vary, such as gaming, viewing films, and so on, none of the research examined alterations after gaming using digital devices.

This study investigated changes the effect of gaming using an iPad, on the ocular accommodative parameters.

## Subjects and Methods

This experimental study was conducted among the university students for a duration of six months. All participants provided written informed consent following a thorough explanation of the study’s nature and potential risks. This study followed the principles outlined in the World Medical Association Declaration of Helsinki. Approval for the experimental protocol and consent procedures was granted by the Institutional Review Board of Visifert Eye Hospital (VEH/IRB/01/0012). The inclusion criteria were best corrected LogMAR distance visual acuity of at least 0.0, N6 at 40 cm using a near English reading chart, and no history of asthenopia. All those with manifest strabismus, systemic or ocular abnormality, or a history of eye surgery were excluded.

### Subjects

A total of 108 individuals received comprehensive eye examinations, of which 80 were chosen as the study’s primary subjects. The remaining 28 were excluded because they did not meet the inclusion criteria. In this study, subjects were chosen using purposive sampling. The subjects in this study were between the ages of 18 and 29. The comprehensive eye evaluation included a visual acuity test for distance using a Log MAR distance visual acuity chart (4 m) and for near an English chart with N notation at 40 cm, along with slit-lamp evaluation.

The subjects underwent a binocular vision evaluation that included the following: Near Point of Accommodation (NPA), Near Point of Convergence (NPC), Negative and Positive Fusional Vergence Amplitudes (NFV, PFV), Accommodative Facility (AF), Vergence Facility (VF), Negative relative accommodation (NRA), Positive relative accommodation (PRA), and Accommodative Response (MEM). The results were compared with age matched normative values (Scheiman and Wick, 2008). All the assessments were conducted with the participant wearing full refractive correction.

### Experimental setup

During the experiment, all the subjects were seated comfortably in chairs, and the brightness level of the room was established using data from a study by Padavettan *et al*. (2021). The intensity of the brightness was 480–500 lux, acquired by LED lights, and no glare from the window was permitted. An iPad mini was used, which has an 8.3-inch Liquid Retina display, 2266 × 1488 resolutions at 326 pixels per inch, wide color display P3, 520 nits (peak) was utilized for the experiment. The display’s illumination was maintained at 460–480 cd/m2. The subjects were assigned to play a ‘Shooting game,’ a sports themed game for 30 minutes. In every five minutes interval the distance was measured by the primary investigator.

### Evaluation of accommodative parameters

#### NearpPoint of accommodation

The NPA is defined as the point nearest to the eye where a target is strongly focused on the retina and is measured using the RAF Rule. Three measurements were obtained in quick succession and average value were noted. The centimeter measurement was converted to diopter to find out the amplitude of accommodation. The average amplitude of each participant was calculated using Hofstetter’s formula (Sterner *et al*., 2004).

#### Negative and positive accommodation

The NRA is an assessment of the maximal capacity to relax accommodation while preserving clear, single binocular vision. The PRA is an assessment of the maximal capacity to stimulate accommodation while retaining clear, single binocular vision. The relative accommodation was assessed using minus (negative) and plus (positive) lenses. The results were obtained in Diopter (D) (Abraham, Srinivasan and Thomas, J. 2015; García, Cacho and Lara, 2002).

#### Accommodative response

The accommodative response was objectively assessed using dynamic retinoscopy by MEM (Monocular Estimation Method). It is an objective means of assessing accommodative response when the patient is actively accommodating. It was carried out with the patient comfortably seated and wearing the necessary refractive correction at usual reading distance and adequate room lighting. The retinoscope head was fitted with a tiny MEM card carrying words or graphics. Participants were instructed to read aloud with both eyes open, and each eye was subjected to dynamic retinoscopy. The quantity of ‘with’ and ‘against’ motion was neutralized using plus lens for ‘with motion,’ while the minus lens was used to for ‘against-the-motion.’ It signifies how much neutralizing lens is used. The lead or lag of accommodation was the difference between the accommodating stimulus and the accommodative response (Leat, 1996; Yekta *et al*., 2017).

#### Accommodative facility

The capacity of the eye to concentrate on stimuli at various distances and in varied sequences in a particular duration is tested using flippers of +/–1.50 D (García *et al*., 2000; Tassinari, 2002).

### Statistical Analysis

All the data from the case sheet was entered into the MS Excel sheet (2019). The data set was analyzed using SPSS (IBM Statistical Package for Social Science Version 26.0). Descriptive statistics were used for the overall analysis. Due to sample size of more than 50, the Kolmogorov-Smirnov test was employed to ensure the normality of the data (Mishra *et al*., 2019). As the data was non-parametric, a non-parametric test was utilized to compare the data, i.e., before vs post activities. The Wilcoxon Signed Rank Test was performed to determine the level of significance of the accommodative parameters. The alpha error was set at 5%.

## Results

The mean age of the 80 participants was 22.96 ± 2.23 years (range from 18–29 years), with 50 males and 30 females. The right eye mean spherical equivalent refraction was –0.71 ± 1.73 diopters (range from +1 to –8 DS), whereas the left eye was –0.71 ± 1.75 diopters (range from +1 to –9 DS).

### Near point of accommodation

**Figure 1:**
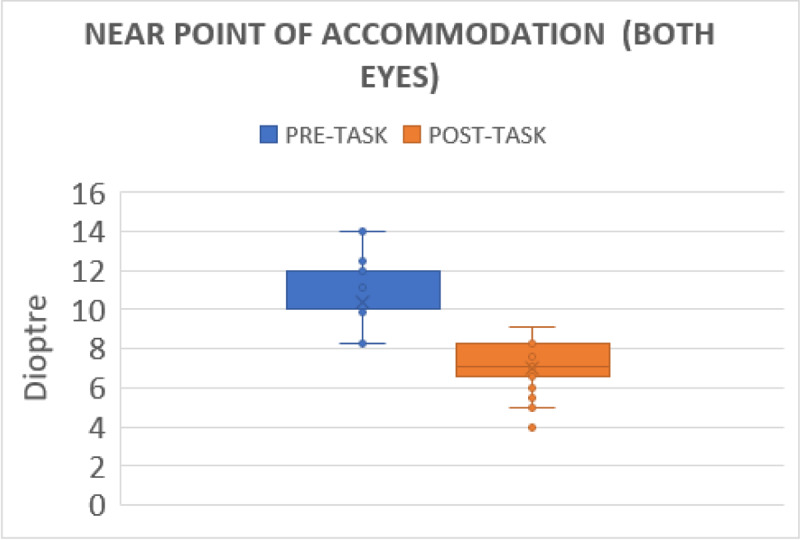
Shows the changes in near point of accommodation for both eyes before and after gaming.

The mean pre-gaming amplitude of accommodation for the right eye, left eye, and both eyes were 10.75D ± 1.83D, 10.60D ± 2.01D, 10.35D ± 1.52D, these values decreased post gaming to 7.15D ± 1.25D for the right eye, 7.24D ± 1.15D for the left eye, and 7.06D ± 1.11D for both eyes (p < 0.001) (Figure 1).

#### Negative relative accommodation

**Figure 2:**
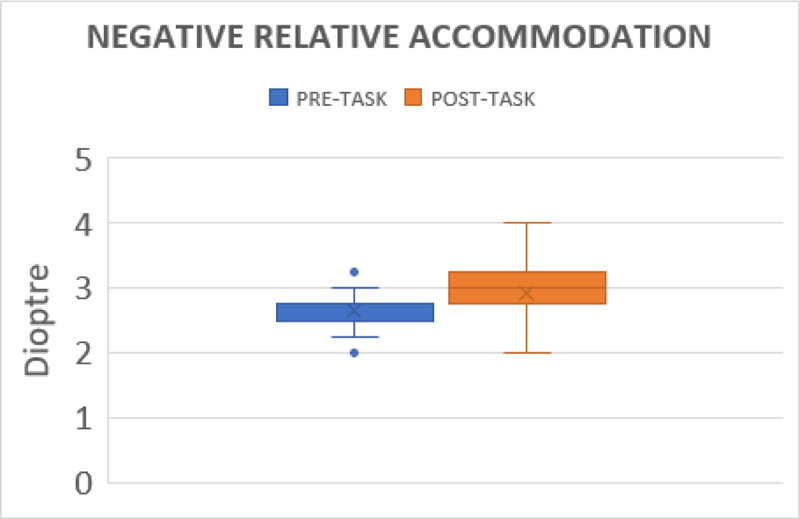
Illustrates the changes in negative relative accommodative before and after gaming.

The NRA before gaming was 2.64 ± 0.23 DS (range from 2.25 to 3.25), but it increased to 2.92 ± 0.37 DS after the task (range from 2.50D to 3.75D) (p < 0.001) (Figure 2).

#### Positive relative accommodation

**Figure 3:**
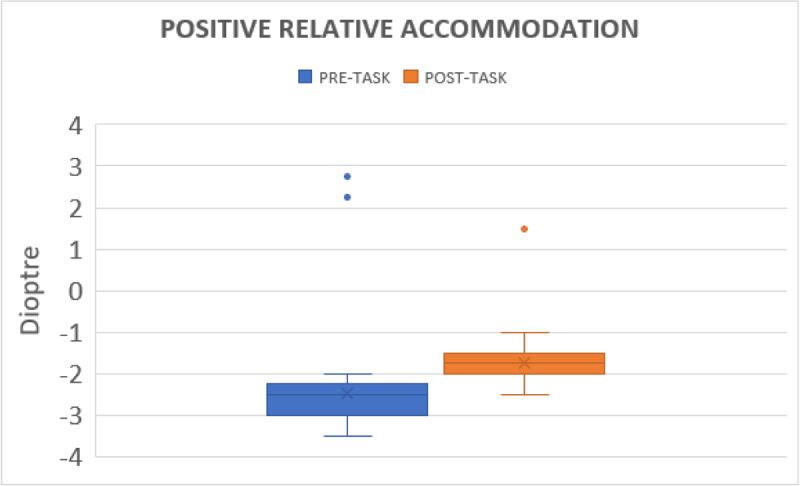
Shows change in positive relative accommodation before and after gaming.

Before gaming, PRA was –2.46 ± 0.41 DS (range from –2.00 DS to –3.50 DS); after the task, it was reduced to –1.78 ± 0.31 DS (range from –1DS to –2DS) (Figure 3).

#### Accommodative response (MEM)

**Figure 4:**
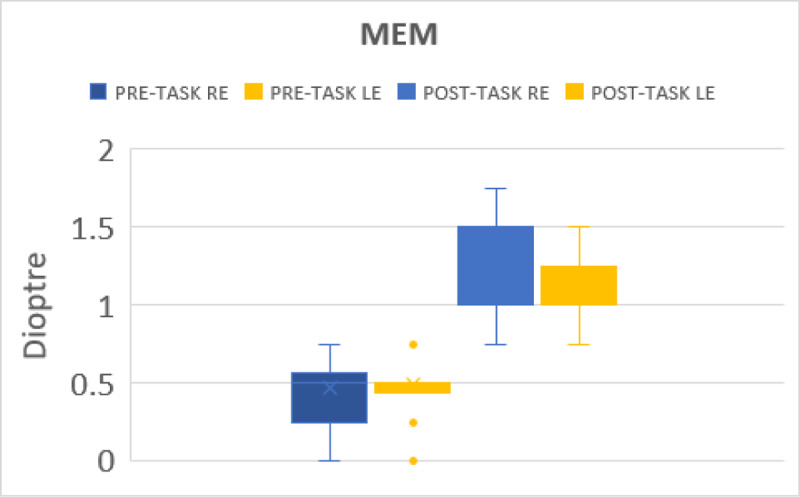
Shows the changes in accommodative response before and after gaming.

The MEM value before gaming were 0.46 ± 0.15 DS and 0.48 DS ± 0.16 DS for right and left eye respectively while the mean MEM post-task was 1.24DS ± 0.26 DS and 1.19 ± 0.24 DS for right and left eye respectively. Both values had a significant change (p < 0.001) (Figure 4).

#### Monocular accommodative facility (MAF)

**Figure 5:**
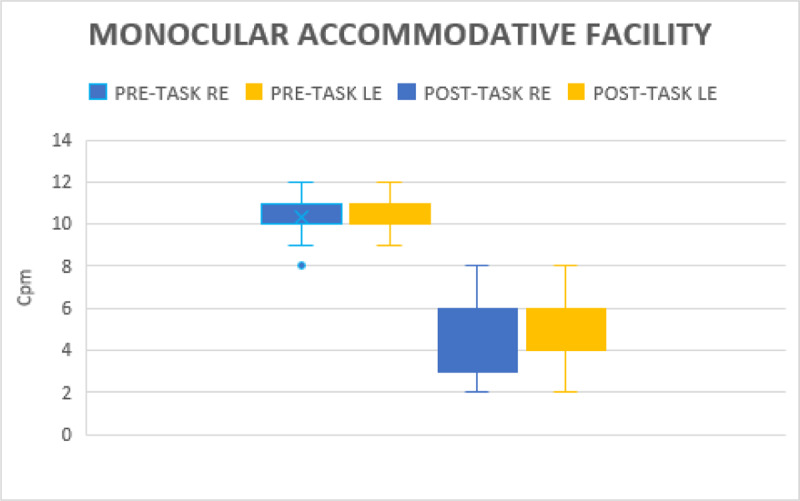
Illustrates Monocular Accommodative facility changes before and after gaming.

Before gaming the MAF for the right eye was 10.34 ± 0.88 cpm and for the left eye, MAF was 10.36 ± 0.82 cpm. MAF for the right eye decreased to 4.54 ± 1.69 cpm after gaming (p < 0.001) and for the left eye to 4.97 ± 1.37 cpm (p < 0.001). This indicated the decreased ability for monocular stimulation and relaxation of ocular accommodative facility (Figure 5).

#### Binocular accommodative facility (BAF)

**Figure 6:**
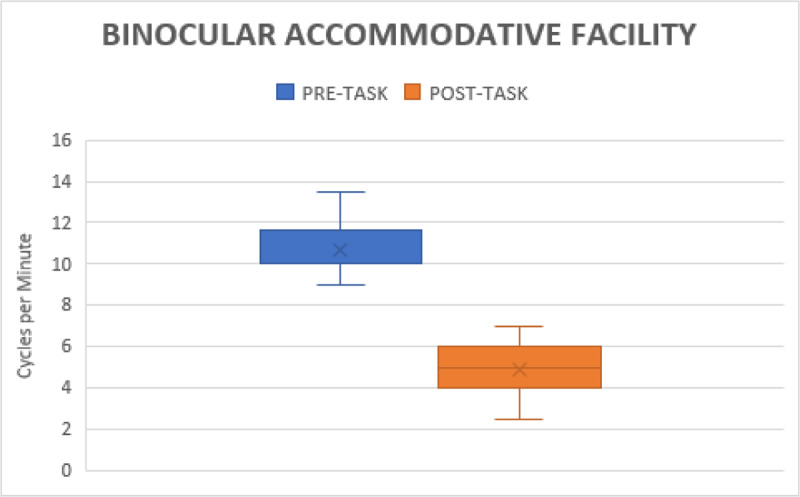
Shows the changes in binocular accommodative facility before and after gaming.

After gaming, BAF decreased to 4.90 ± 1.06 cpm from 10.65 ± 0.98 cpm (p < 0.001) (Figure 6).

All results are summarized in Table 1.

**Table 1:** Mean and standard deviation of ocular accommodative parameters before and after gaming using VDU.


PARAMETERS	EYE	PRE TASK		POST TASK		P VALUE

		MEAN	SD	MEAN	SD	

MEM	RE	0.46	0.19	1.24	0.26	<0.001

	LE	0.48	0.16	1.19	0.24	<0.001

NPA	RE	10.75	1.83	7.15	1.25	<0.001

	LE	10.60	2.01	7.24	1.15	<0.001

	BE	10.35	1.52	7.06	1.11	<0.001

NRA		2.64	0.23	2.92	0.37	<0.001

PRA		2.46	0.41	1.78	0.31	<0.001

MF	RE	10.34	0.88	4.54	1.69	<0.001

	LE	10.36	0.82	4.97	1.37	<0.001

BAF		10.65	0.98	4.90	1.06	<0.001


## Discussion

In this current study we evaluated the accommodative parameters before and after 30 minutes of continuous tablet gaming.

Significant changes were observed for the NPA after the gaming phase among the subjects. This may be the result of the tonic accommodation brought on by long-term close activity. Many studies suggest that 30 minutes of reading, watching movies, and computer gaming causes a decrease in NPA, where in our study shows 30 minutes of gaming also causes lower in NPA in healthy adults (Kang *et al*., 2021; Nayak *et al*., 2020; Padavettan *et al*., 2021).

The NRA and PRA were significantly affected after the activity. To maintain binocularity, NRA and PRA both depend on fusional vergence. The decrease in relative accommodation may be influenced by the reduction in fusional vergence. Padavettan *et al*. (2021) reported reduced NRA and PRA after 30 minutes of reading activity. Lee *et al*. (2019) showed a decrease in NRA and PRA after 30 minutes of movie watching on a smart phone. None of the studies addressed the effects of tablet gaming.

Lag of accommodation was noted post-activity, which was similar to the findings of others after near activity (Ha *et al*., 2014; Kang *et al*., 2021; Lee *et al*., 2019; Padavettan *et al*., 2021). The reason for lag of accommodation in our study is likely to be the task of continuous gaming for 30 minutes.

Both BF and MF were significantly affected post-task. It was consistent with other studies among young people, wherein participants were given tablet-based visual tasks to complete after reading and viewing movies (Padavettan *et al*., 2021; Park *et al*., 2014).

## Conclusion

Our study found a significant decrease in accommodative parameters after 30 minutes of gaming using an iPad. Ocular fatigue and non-strabismic binocular vision anomalies may develop earlier in prolonged digital device users as compared to individuals with lesser exposure. To lessen the likelihood of binocular vision anomalies (which might result in asthenopia), regular breaks are often suggested when playing games using digital devices. However, the impact of taking breaks on binocular vision parameters has not been studied and this creates scopes for future studies.

## Data Accessibility Statement

The datasets generated during and/or analyzed during the current study would be available on request for research purposes.
